# PEPI Lab: a flexible compact multi-modal setup for X-ray phase-contrast and spectral imaging

**DOI:** 10.1038/s41598-023-30316-5

**Published:** 2023-03-14

**Authors:** Luca Brombal, Fulvia Arfelli, Ralf Hendrik Menk, Luigi Rigon, Francesco Brun

**Affiliations:** 1 Department of Physics, University of Trieste, 34127 Trieste, Italy; 2 Division of Trieste, National Institute for Nuclear Physics (INFN), 34127 Trieste, Italy; 3 Elettra Sincrotrone Trieste S.C.p.A., 34149 Basovizza, TS Italy; 4 Department of Engineering and Architecture, University of Trieste, 34127 Trieste, Italy

**Keywords:** Imaging techniques, Design, synthesis and processing

## Abstract

This paper presents a new flexible compact multi-modal imaging setup referred to as PEPI (Photon-counting Edge-illumination Phase-contrast imaging) Lab, which is based on the edge-illumination (EI) technique and a chromatic detector. The system enables both X-ray phase-contrast (XPCI) and spectral (XSI) imaging of samples on the centimeter scale. This work conceptually follows all the stages in its realization, from the design to the first imaging results. The setup can be operated in four different modes, i.e. photon-counting/conventional, spectral, double-mask EI, and single-mask EI, whereby the switch to any modality is fast, software controlled, and does not require any hardware modification or lengthy re-alignment procedures. The system specifications, ranging from the X-ray tube features to the mask material and aspect ratio, have been quantitatively studied and optimized through a dedicated Geant4 simulation platform, guiding the choice of the instrumentation. The realization of the imaging setup, both in terms of hardware and control software, is detailed and discussed with a focus on practical/experimental aspects. Flexibility and compactness (66 cm source-to-detector distance in EI) are ensured by dedicated motion stages, whereas spectral capabilities are enabled by the Pixirad-1/Pixie-III detector in combination with a tungsten anode X-ray source operating in the range 40–100 kVp. The stability of the system, when operated in EI, has been verified, and drifts leading to mask misalignment of less than 1 $\upmu$m have been measured over a period of 54 h. The first imaging results, one for each modality, demonstrate that the system fulfills its design requirements. Specifically, XSI tomographic images of an iodine-based phantom demonstrate the system’s quantitativeness and sensibility to concentrations in the order of a few mg/ml. Planar XPCI images of a carpenter bee specimen, both in single and double-mask modes, demonstrate that refraction sensitivity (below 0.6 $\upmu$rad in double-mask mode) is comparable with other XPCI systems based on microfocus sources. Phase CT capabilities have also been tested on a dedicated plastic phantom, where the phase channel yielded a 15-fold higher signal-to-noise ratio with respect to attenuation.

## Introduction

Owing to the advancements in imaging detector technology and the development of robust X-ray optical devices, X-ray phase-contrast imaging (XPCI) and X-ray spectral imaging (XSI) are becoming viable options for compact laboratory systems.

The advantage of XPCI over conventional X-ray imaging originates from the fact that the phase modulation (i.e. phase-shift) imparted by the imaged sample on the impinging X-ray wavefront is—for light materials—two to three orders of magnitude larger than the amplitude modulation that is responsible for conventional attenuation contrast^1,2^. For this reason, XPCI enables the extraction of image contrast from features scarcely visible in conventional attenuation-based imaging, such as soft tissues or highly granular structures^3^. Many XPCI techniques have been pioneered in synchrotron radiation facilities, proving their advantages in a vast range of applications^3,4^. The next compelling goal is to carry out the transition of XPCI from large and expensive infrastructures, to compact and (relatively) cheap setups that can be fitted in a laboratory environment^5^. In this context, together with Talbot-Lau gratings interferometry^6^ and speckle-based imaging^7^, edge illumination (EI)^8^ is one of the most promising XPCI techniques enabling this transition. EI makes use of one absorbing periodic grid (i.e., mask) that structures the X-ray beam impinging on the sample into a series of mutually independent non-interfering beamlets. By analyzing the intensity reduction (attenuation), displacement (refraction), and broadening (diffusion) of each individual beamlet due to the sample, either through a second analyzer mask (double-mask mode^9^) or directly with the detector’s pixel (single-mask/beam-tracking mode^10,11^), EI allows to simultaneously extract three contrast channels, namely attenuation, (differential) phase and dark field, also known as ultra-small-angle scattering or simply scattering^9,12^.

Independently from advancements in XPCI, the introduction of photon-counting hybrid detectors with high-Z sensors and energy-based discrimination capabilities has given an unprecedented boost to the field of X-ray spectral imaging^13,14^. The goal of XSI is to separate and quantify the presence of materials with different chemical compositions within the sample, based on the energy dependence of their attenuation coefficients. In the biomedical imaging field, the most well-known application of XSI is arguably the quantification of purposely injected contrast agents (e.g., iodine, barium, gadolinium), targeting specific anatomical/functional districts^15,16^. In XSI two (or more) images with different energy content are required; historically, this has been accomplished either by using X-ray sources producing two different X-ray spectra (dual-energy) or by employing layered detectors where the first layer is sensitive to soft X-rays while the second is sensitive to the harder component of the spectrum (dual-layer). On the other hand, spectral detectors feature energy-calibrated and software-controlled thresholds, that enable the discrimination of X-rays into two (or more) energy bins upon their detection in a single shot^17,18^. This brings to a great simplification of the system in terms of hardware and allows targeting different applications simply by changing the energy thresholds.

In this framework, the development of flexible imaging systems enabling X-ray imaging with multiple modalities, such as XPCI and XSI, seems to be a promising, yet not fully explored research field. A brilliant example of an extremely flexible imaging system has been provided by Samber et al.^19^, who demonstrate the integration of 10 different scanning techniques, ranging from laminography to time-resolved 4D imaging, and also comprising propagation-based phase contrast. Interesting examples of XPCI and detector-based XSI integration have been recently provided by Ji et al.^20^ and Braig et al.^21^ in the context of grating interferometry and by Vazquez et al.^22^ in the field of propagation-based imaging. Moreover, despite not making use of the detector’s spectral capabilities, several XPCI systems employing photon-counting detectors have been documented in literature ranging from breast CT applications at synchrotrons^23^ to small animal imaging in the laboratory^24^.

In this work, a novel multi-modal X-ray system specifically targeting the integration of edge-illumination and spectral imaging is presented. The setup, referred to as PEPI Lab, has been developed in the framework of the PEPI (Photon-counting Edge-illumination Phase-contrast Imaging) project and pursues two key objectives, namely compactness and flexibility. The goal of compactness has been reached through a design aimed at minimizing the footprint of motion stages in the X-ray path while retaining the adequate number of degrees of freedom for X-ray optics alignment, motion, and sample rotation in CT scans. The system flexibility is ensured by the custom design of the masks’ holders and by dedicated motion stages whereby masks and samples can be inserted or removed from the X-ray beam without requiring hardware modification, re-alignment or sample repositioning. In this way the system can accommodate: i) photon-counting imaging setup—to be used for conventional or preliminary sample assessment (scout) imaging; ii) spectral imaging setup (detector in two-thresholds/colors mode)—to be used when attenuation-based material decomposition is required; iii) double-mask EI phase-contrast setup—to be used when sensitivity to all contrast channels (attenuation, refraction, dark-field) is required; iv) single-mask EI phase-contrast setup—to be used when sensitivity to attenuation and refraction only is required; Moreover, the highly absorbing masks can be used in a wide energetic range from soft (40 kVp) to hard (100 kVp) X-ray spectra, thereby being suitable for both soft-tissue and material science samples. Spectral performances are ensured by the photon-counting Pixirad-1/Pixie-III detector^25^ that, thanks to the high efficiency of its CdTe sensor and to the on-chip charge-sharing compensation mechanisms, allows applications making use of several contrast media in a wide energetic range^26^. Considering its characteristics, PEPI Lab can be regarded as a rather general-purpose X-ray imaging platform for samples on the centimeter scale, that can be further specialized targeting specific applications ranging from e.g., ex-vivo small animal or biological tissue samples, to additive manufacturing products or quantitative material characterization.

## Experimental setup

The implementation of the PEPI Lab required several steps from the definition of the system requirements to the hardware and control software development. As all these steps are common in the development of most imaging systems, they are detailed in the present section following the setup construction from the ground up.

**Figure 1:**
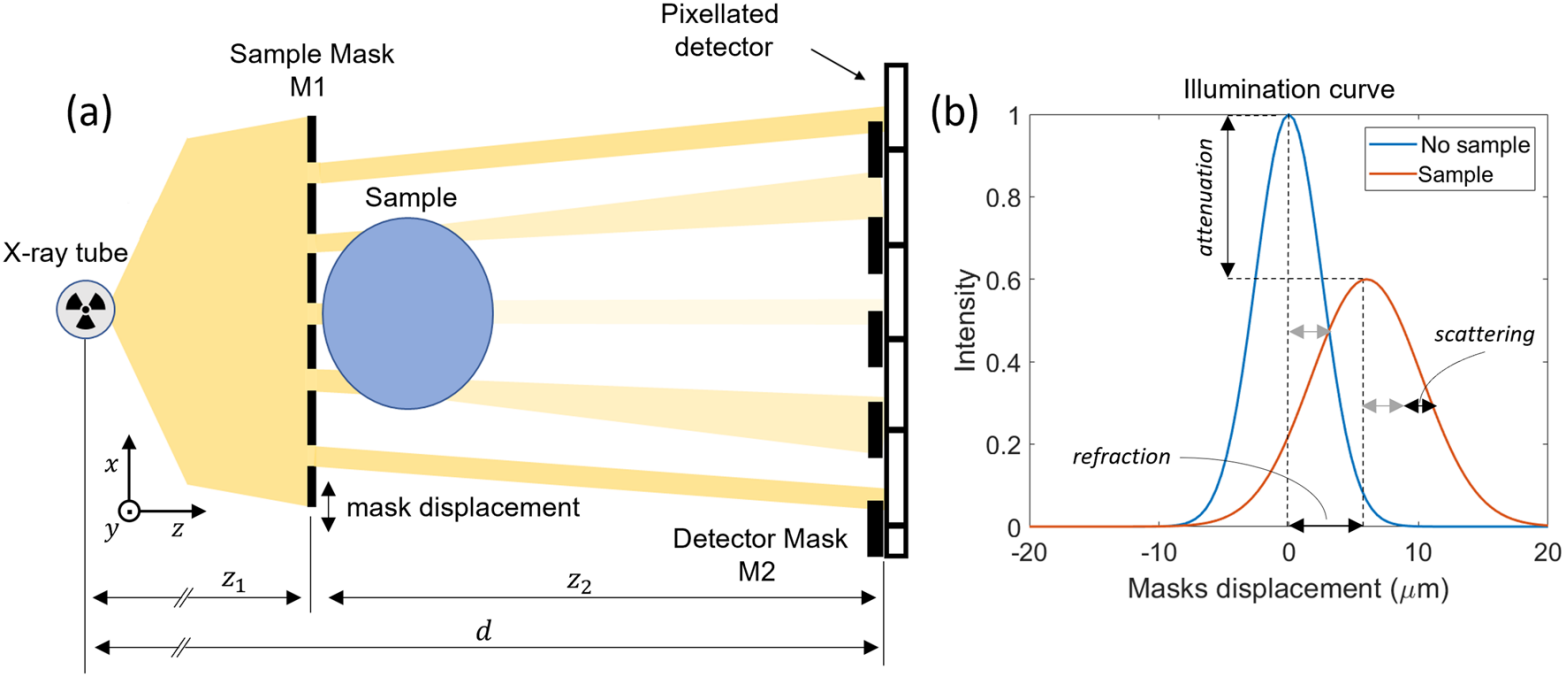
In (**a**) a sketch (not to scale) of a double-mask EI setup: due to the sample each beamlet is reduced in intensity (attenuation), deflected (refraction) and diffused (dark field). In (**b**) the characteristic illumination curves obtained by displacing the masks without (light blue) and with (orange) the sample in place.

### Edge illumination

The original and most common EI implementation is the so-called double-mask setup^8^. As sketched in panel (a) of Fig. 1, this arrangement makes use of one periodic absorbing structure (sample mask, M1) positioned upstream from the sample, that structures the impinging beam into an array of mutually independent narrow beams, referred to as beamlets. Each beamlet is intercepted by a second periodic structure (detector mask, M2) scaled by the geometrical magnification factor with respect to M1. As a function of the relative displacement of the two masks, typically performed by moving M1, each pixel experiences an intensity variation that is referred to as the illumination curve (IC). The presence of the sample modifies the IC by reducing its amplitude due to attenuation effects, shifting its center due to refraction effects (i.e. differential phase-contrast), and broadening its width due to scattering effects (i.e. dark field), as depicted in panel (b) of Fig. 1. By sampling the IC on three or more points (i.e. stepping procedure) both with and without the sample, all these effects can be uncoupled therefore producing three images containing complementary information^9^. The IC shape is a key feature of an EI setup, as it ultimately determines its sensitivity to phase effects. Focusing on the refraction signal, it can be demonstrated analytically that the signal-to-noise ratio follows^27^:

1 $$\begin{aligned} SNR_{ref} \propto \frac{z_2\sqrt{I_0}}{M}\frac{IC'}{\sqrt{IC}} \end{aligned}$$

where $z_2$ is the sample-mask to detector distance (see Fig. 1), *M* is the geometrical magnification, $I_0$ is the photon flux without the sample in place and at the peak of the illumination curve, $IC'$ and *IC* are the illumination curve’s slope and working point, respectively.

Another advantage of EI is that the spatial resolution is primarily determined by the M1 aperture size as it defines the size of the beamlet. On the other hand, in order to reach a complete illumination of the sample, and thus an M1 aperture limited spatial resolution, the sample must be displaced with respect to M1 in sub-mask period increments, a procedure referred to as dithering^28^. As a first approximation, if the M1 period is $p_1$ and the aperture is $a_1$ then the number of dithering steps should be in the order of $p_1/a_1$ to ensure adequate object’s sampling.

### Required imaging modalities

The presented tabletop setup is flexible, i.e. it allows the implementation of multiple imaging modalities, whereby the switch to any modality is software controlled and requires no hardware modifications.

**Figure 2:**
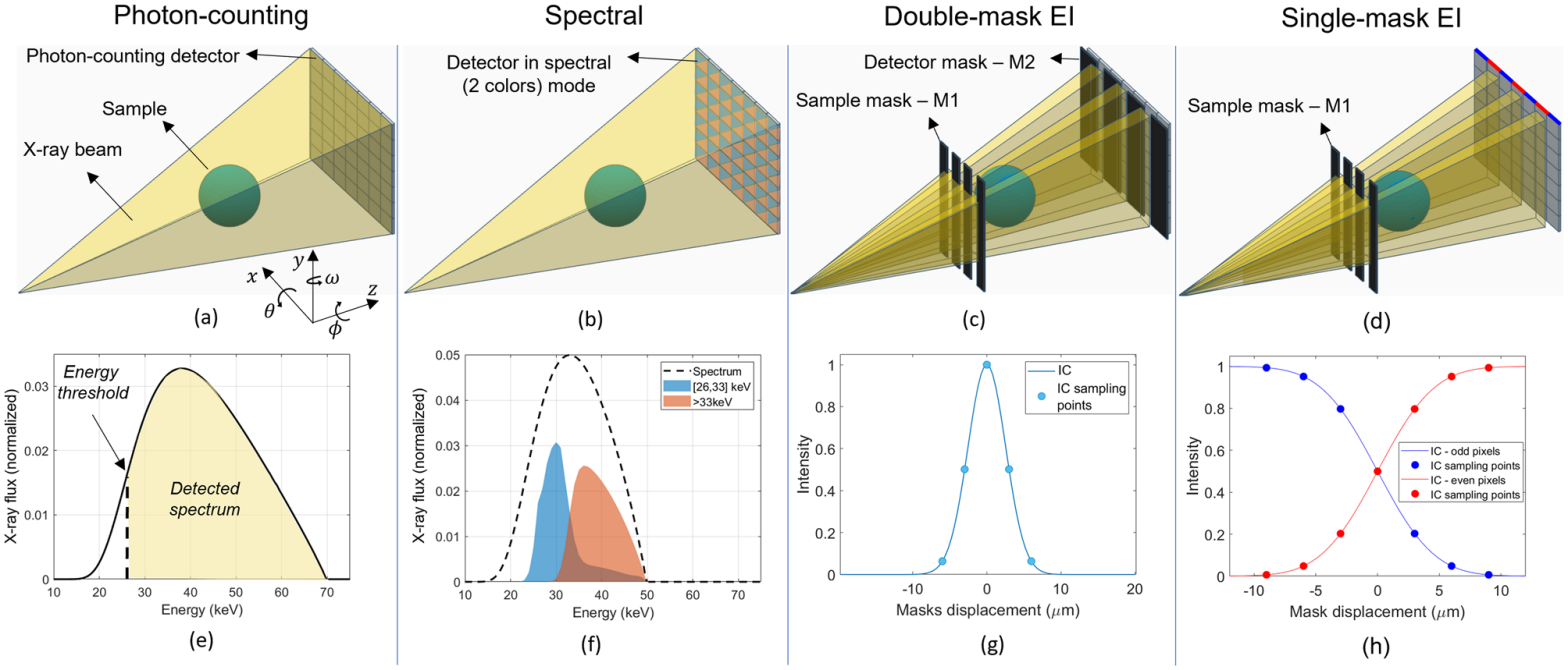
The four imaging modalities, namely photon-counting (**a**), spectral (**b**), single-mask EI (**c**), and double-mask EI (**d**). In (**e**) a typical X-ray spectrum used in photon-counting CT, in (**f**) the spectral response of the Pixirad-1/Pixie-III over the 2 selected energy bins optimized for the iodine K-edge (filled plots), expressed in counts per input photon per keV, along with the X-ray spectrum (dashed line). X-ray spectra are area normalized. In (**e**) and (**f**) the typical IC shapes for the single and double mask arrangements, respectively.

Specifically, the system must allow for photon-counting and quantitative XSI imaging both in planar and tomographic geometries at a scale of a few tens of micrometers. The difference between these two modalities is in the detector operation mode, where only one energy threshold is selected for photon-counting imaging, see panels (a) and (e) of Fig. 2, while two thresholds combined with the charge sharing recovery are selected in XSI, see panels (b) and (d) of Fig. 2. In both modalities, no masks are present in the X-ray beam path, while adequate beam filtration should be provided in order to shape the X-ray spectrum limiting beam-hardening effects and optimizing the spectral decomposition^26^. The latter step also requires a careful characterization of the detector spectral response^29,30^.

The system is required to accommodate the double-mask EI imaging (both planar and tomographic), as depicted in panels (c) and (g) of Fig. 2, without the need for manual mounting procedures. Additionally, since the alignment of the masks is typically a lengthy procedure, minimal misalignment should be introduced when switching modalities. To reach this goal, the integration of motion stages with good position repeatability, sufficiently long travel range, and sufficiently stiff mounting supports is crucial. Moreover, for the sake of compactness, the footprint of motion stages and supports must be reduced as much as possible.

An additional imaging modality referred to as single-mask EI^10^, see Fig. 2d, can be implemented in the system. This technique makes use of the sole sample mask, whose pitch is selected to project a beamlet every other pixel column (i.e. skipped geometry). By displacing the mask with respect to the detector pixel matrix, an intensity modulation conceptually equivalent to the IC in the double-mask arrangement is registered, and a complementary behavior of odd and even pixel columns is observed, i.e. a decrease of intensity in odd pixels corresponds to an increase in even pixels and vice-versa, as depicted in Fig. 2h. By shooting the beamlets exactly in between two pixels a uniform illumination is reached. When in the beam, the sample locally perturbs this uniformity by reducing the overall intensity due to attenuation and creating an intensity unbalance between even and odd pixels due to refraction, i.e. beamlet deviation^31^. By treating this problem as a linear system inversion with two inputs, even and odd pixels intensity, and two outputs, attenuation and refraction, the single-mask arrangement can yield the two parametric images in a single shot. Single-mask is complementary to double-mask EI since it does not require sample mask motion during acquisition, therefore being a single-shot technique. On the other hand, single-mask does not allow for a straightforward extraction of the dark-field signal, thus providing limited information compared to double-mask EI.

### Simulation-based EI system design

The design of the EI system and the final hardware specifications relied on simulation results produced with a dedicated Monte-Carlo (MC) simulation platform based on the Geant4 toolkit whereby X-ray refraction effects have been included^32^. As demonstrated by a number of recently published studies, MC approaches using robust and widespread simulation engines are becoming popular among the XPCI community^33–36^. The main advantage of MC over simpler analytical or hybrid solutions is the possibility of including many realistic experimental details, ranging from the actual spatial and spectral distributions of the X-ray source to the detector response, in a single and comprehensive simulation environment. On the other hand, MC simulations are typically computationally intensive and more time-consuming with respect to other approaches^32^.

**Figure 3:**
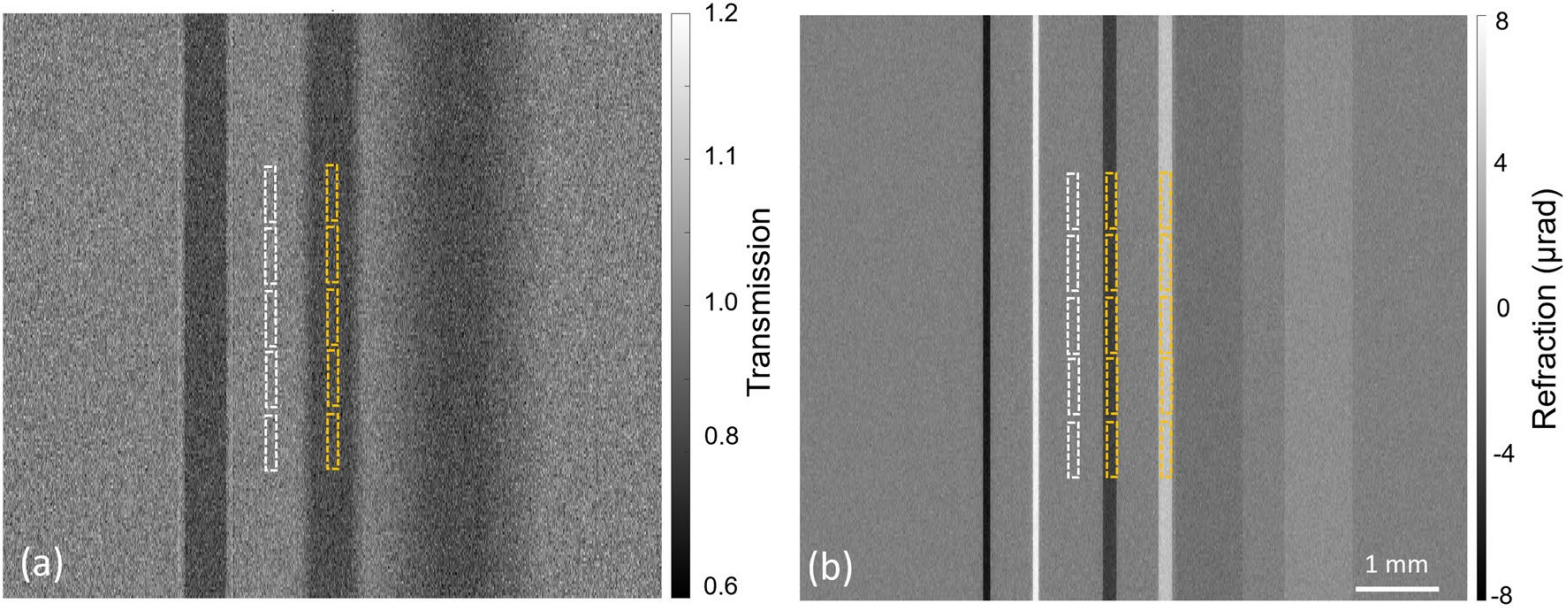
Examples of simulated images after attenuation (**a**) and refraction (**b**) signal extraction. Samples are three adjacent PMMA trapezoidal prisms with different inclinations^30^. The yellow and white dashed rectangles are the signal and background ROIs, respectively, which are used for SNR computation.

Concerning the PEPI imaging system, many hardware parameters that cannot be modified once the equipment is purchased (e.g., masks, X-ray tube) were tested through MC simulations, therefore guiding the choice of instrumentation. Specifically, a double-mask EI setup including all the related mask and sample movements has been simulated^30^ to study the effects of X-ray tube focal spot size, system dimension and geometry, mask aspect ratio and substrate composition. The choice of a double-mask simulation is justified by the fact that this configuration is more commonly used with respect to the single-mask arrangement. The simulated sample is made by three PMMA trapezoidal prisms with different inclinations^30^. Each simulated acquisition consists of 2 steps on the IC (scattering signal is neglected) and 8 dithering steps, and a total of 2$\times$10^9^ X-ray photons per step. Simulated data are processed to uncouple attenuation and refraction^32^ channels, as depicted in Fig. 3. From the final images, both attenuation and refraction signal-to-noise ratio (SNR) are computed. Consistently with previous publications^10,30^, in attenuation images the SNR is computed as $|I_d - I_b|/\sigma _b$ where $I_d$ and $I_b$ are the transmission values measured on the detail and background, respectively, while $\sigma _b$ is the standard deviation of the background. In refraction images the SNR is defined as $(|I^+_d| + |I^-_d|)/2\sigma _b$ where $I^+_d$ and $I^-_d$ are the positive and negative refraction signals corresponding to the 2 inclined edges of the sample. When considering refraction images, $\sigma _b$ is also generally regarded as a measure of angular sensitivity^27^. SNR measurements are repeated for each image on 5 pairs of non-overlapping ROIs. Mean SNR values are used as the metric to evaluate the impact of the system’s parameters on image quality. Uncertainties are computed as the standard deviation over the five measurements.

**Figure 4:**
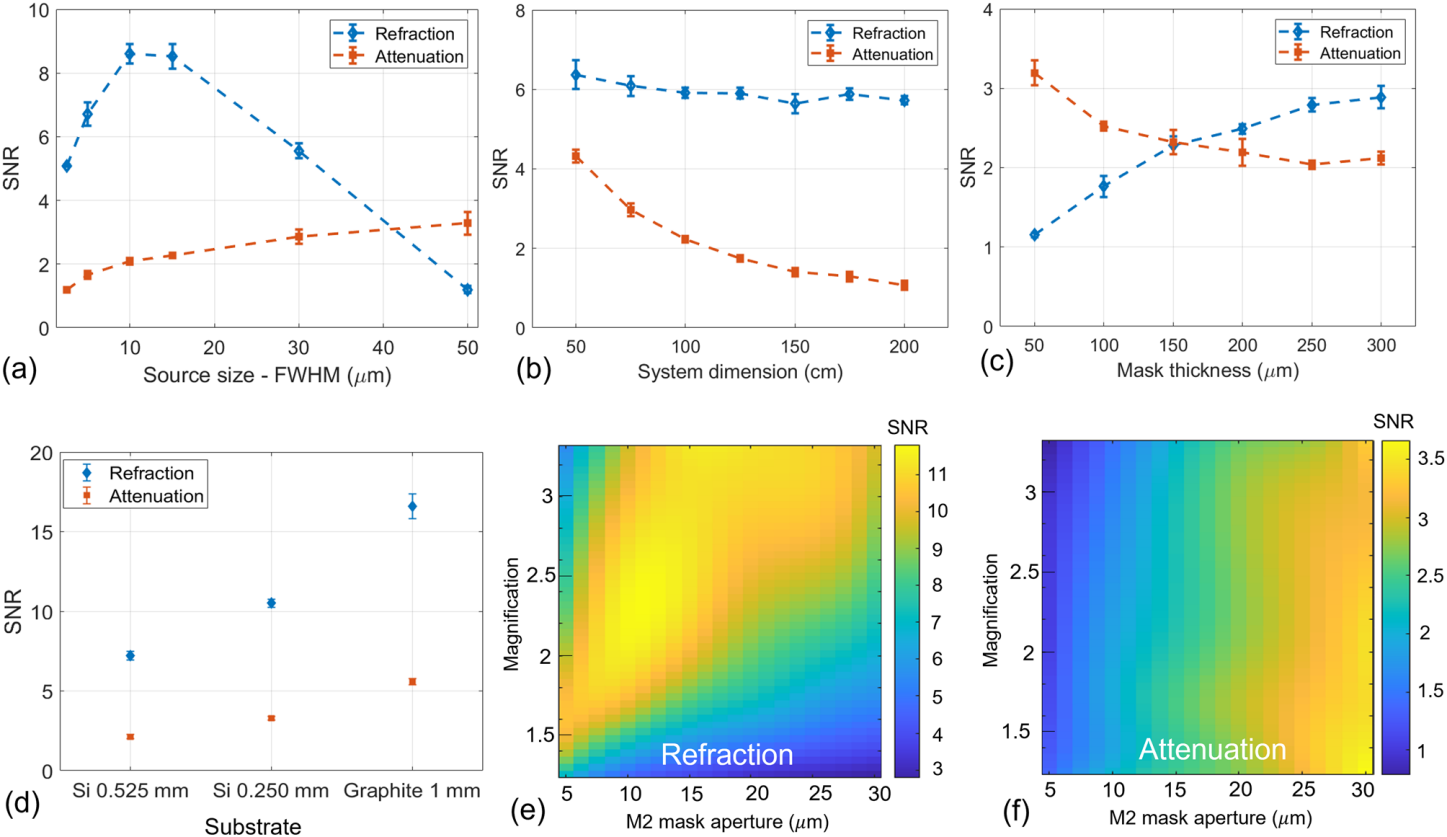
Refraction and attenuation SNR measured from simulated images as a function of the X-ray source size (**a**), the overall system dimension (**b**), the mask thickness (**c**) and the mask substrate (**d**), M2 mask aperture and magnification (**e**,**f**).

#### X-ray tube focal spot size

In principle, EI only requires the projected source size to be smaller than the mask pitch, in order to avoid crosstalk between adjacent beamlets. Nonetheless, the source size affects the shape (i.e. width) of the IC, thus being a key factor in the definition of the system’s sensitivity to phase effects, as shown in Eq. (1). Specifically, if the projected source size is smaller than the mask aperture, the IC width will be mainly determined by the mask aperture while, in the opposite case, it will be defined by the source extension.

In general, a sharper IC leads to a higher refraction sensitivity, meaning that a given beamlet’s refraction is converted to a larger signal modulation. From this consideration, it seems that small source sizes should always be preferred to boost phase sensitivity. On the other hand, small focal spots, of the order of a few microns, can be achieved only in low-power sources, of the order of a few watts. This in turn leads to a lower X-ray flux, bringing to an increase of statistical noise, i.e. a decrease in SNR, for a given exposure time. In order to evaluate the optimal trade-off between these counteracting effects, simulations at different focal spot sizes, in the range from 2.5 to 50 $\upmu$m (Gaussian distribution, FWHM), have been performed at a fixed magnification of 2 and a detector mask aperture of 15 $\upmu$m, the latter being a typical value for EI systems that usually employ apertures in the 10 to 20 $\upmu$m range^10,24^. To take into account the effect of the source size on the flux, a linear relationship between focal spot size and flux has been considered, as commonly suggested by X-ray tube manufacturers, and verified experimentally in the laboratory.

Results, expressed in terms of SNR as a function of the focal spot size, are shown in panel (a) of Fig. 4. From the plot, it can be seen that the SNR for the refraction images reaches a maximum at around 10 to 15 $\upmu$m, which corresponds to the mask aperture. Moreover, the refraction SNR has a less steep degradation when moving towards larger focal spot sizes with respect to smaller focal spot sizes. On the other hand, the attenuation SNR, which is mainly driven by the statistics regardless of the IC shape, is monotonically increasing with the focal spot size, hence with photon statistics.

#### System dimension

The system dimension *d*, i.e. the source-to-detector distance, is a crucial parameter when pursuing compactness. At a fixed magnification, a larger system dimension is related to a larger distance between the sample and the detector $z_2$. Following Eq. (1), this brings to a higher refraction SNR due to the fact that, for a given refraction angle, the lateral displacement of the beamlet is proportional to $z_2$. On the other hand, a larger source-to-detector distance leads to a decrease in the flux, therefore proportionally increasing image noise, and decreasing the SNR. To study these two effects, simulations with different system dimensions, ranging from 0.5 to 2.0 m, have been performed at fixed magnification and statistics. As predicted by theory and suggested by experimental data obtained with other EI systems^37^, simulation results in Fig. 4 panel (b) show that the two effects cancel out, bringing to a constant refraction SNR for all the investigated system dimensions. On the other hand, analogously to the findings of the previous section, the attenuation SNR is only sensitive to the X-ray flux, therefore monotonically decreasing at larger system dimensions.

While not affecting the refraction SNR, it should be mentioned that the system dimension affects the refraction dynamic range, i.e. the largest detectable refraction angle before cross-talk between neighboring beamlets sets in. In fact, by defining the maximum detectable refraction $\alpha _{max}$ as the angle that shifts one beamlet by exactly one mask pitch $p_2$, we get:

2 $$\begin{aligned} \alpha _{max} = \frac{p_2}{z_2} = \frac{p_2}{d}\frac{M}{M-1} \end{aligned}$$

where the identity $z_2 = d (M-1)/M$ has been used. From the previous equation it is clear that, at fixed magnification, the dynamic range is inversely proportional to the system dimension.

Overall, within the limitations imposed by the footprint of sample and instrumentation (e.g., motion stages) and when no radiation dose constraints are present, compact systems are preferable since they bring increased attenuation SNR and dynamic range, without affecting the refraction SNR.

#### Masks thickness and substrate

In EI, the mask septa must ensure a high absorption (ideally, 100%) to avoid cross-talk between adjacent beamlets, and, to this end, high-Z metals, e.g. gold, are employed. Masks are typically produced via the X-ray LIGA process^38^, yielding high aspect ratio metallic structures on low-Z substrates such as silicon or graphite. In principle, when considering a parallel beam irradiation geometry, the thickness of the metallic septa can be chosen as large as possible to avoid transmission. On the other hand, having thick septa poses a two-fold challenge; thicker septa require a thicker substrate, therefore bringing a loss in the X-ray flux transmitted through the apertures. Moreover, in cone beam irradiation geometry (as in laboratory compact systems), thick masks cause a strong penumbra effect^32,39,40^, drastically reducing the flux of photons at a large lateral emission angle unless a bent mask geometry is adopted^41^. For these reasons, both mask thickness and substrate must be optimized.

Since the implemented setup should withstand X-ray spectra up to 100 kVp, simulations have been performed considering a 100 kVp spectrum and by varying the thickness of the gold septa in the range from 50 to 300 $\upmu$m at 50 $\upmu$m increments. From the results reported in Fig. 4 panel (c), it can be seen that the refraction SNR increases for thicker masks, reaching a plateau above 250 $\upmu$m where mask absorption is around 90%. An opposite behavior is observed for the attenuation SNR. This is due to the fact that thin masks allow partial transmission, therefore effectively increasing the photon flux and decreasing the attenuation image noise. In order to limit as much as possible the penumbra effect, a mask thickness of 250 $\upmu$m has been selected for the experimental setup, as it is the smallest value ensuring an optimal refraction SNR. It should be remarked that thicknesses above 300 $\upmu$m have not been simulated as the penumbra would have caused a total absorption of X-rays in the periphery of the field of view (along the horizontal direction).

Once the mask thickness has been defined, three different substrates, corresponding to the options given by the masks’ manufacturer, have been simulated, namely 1 mm of graphite, 0.525 mm, and 0.250 mm of silicon. Since substrates mainly impact the transmitted low-energy X-rays flux, simulations have been performed at a voltage of 40 kVp and with a small filtration of 0.1 mm of aluminum. Results in Fig. 4 panel (d) show that the substrate choice has a major impact on the final image SNR, both for attenuation and refraction channels. Specifically, the 1 mm graphite option yields the highest SNR (more than 2 times higher than 0.525 mm silicon), being the preferred choice for the new setup. In general, it should be noted that the silicon substrate is typically the cheapest option and, for a given thickness, silicon is stiffer than graphite, so it might be preferable in applications disregarding low-energy photons.

#### Masks aperture and position

As shown in Eq. 1, the refraction SNR is linked to the system’s magnification, to the slope of the illumination curve, and to the flux on the detector, where the latter two terms are strongly dependent on the mask aperture. For this reason, it is reasonable to carry out the optimization of the system magnification and mask aperture jointly. In panels (e) and (f) of Fig. 4, the SNR as a function of magnification and M2 mask aperture is shown, respectively for refraction and attenuation, where the aperture varies between 5 and 30 $\upmu$m and the magnification between 1.25 and 3.33. Simulations are carried out at a fixed system dimension of 100 cm and source size of 5 $\upmu$m, where this value was chosen as it is the typical smallest focal spot value for many commercial microfocus X-ray sources. From the surface plots, it is clear that higher magnifications, coupled with larger M2 mask apertures, are generally preferable in terms of refraction. On the other hand, the behavior of attenuation SNR is entirely determined by the mask aperture, where larger apertures lead to higher SNR, as the X-ray flux is increased. In addition to SNR optimization, it should be remarked that, when choosing the system magnification, other two practical factors must be considered, namely the field-of-view (inversely proportional to magnification) and the penumbra effect due to the finite mask thickness (directly proportional to magnification and inversely proportional to mask thickness). For this reason, with the aim of having a reasonable field-of-view in the order of 15 mm to fit commonly used sample containers such as Falcon tubes, and considering the detector’s sensor lateral dimension of around 30 mm, a mask magnification around 2 has been selected for the implemented setup. Concerning the M2 mask aperture, a value of 19 $\upmu$m has been chosen as it is the value closer to the simulated maximum that guarantees that the intensity reduction due to the penumbra effect does not exceed 50% when working on the slopes of the IC.

### Hardware

**Figure 5:**
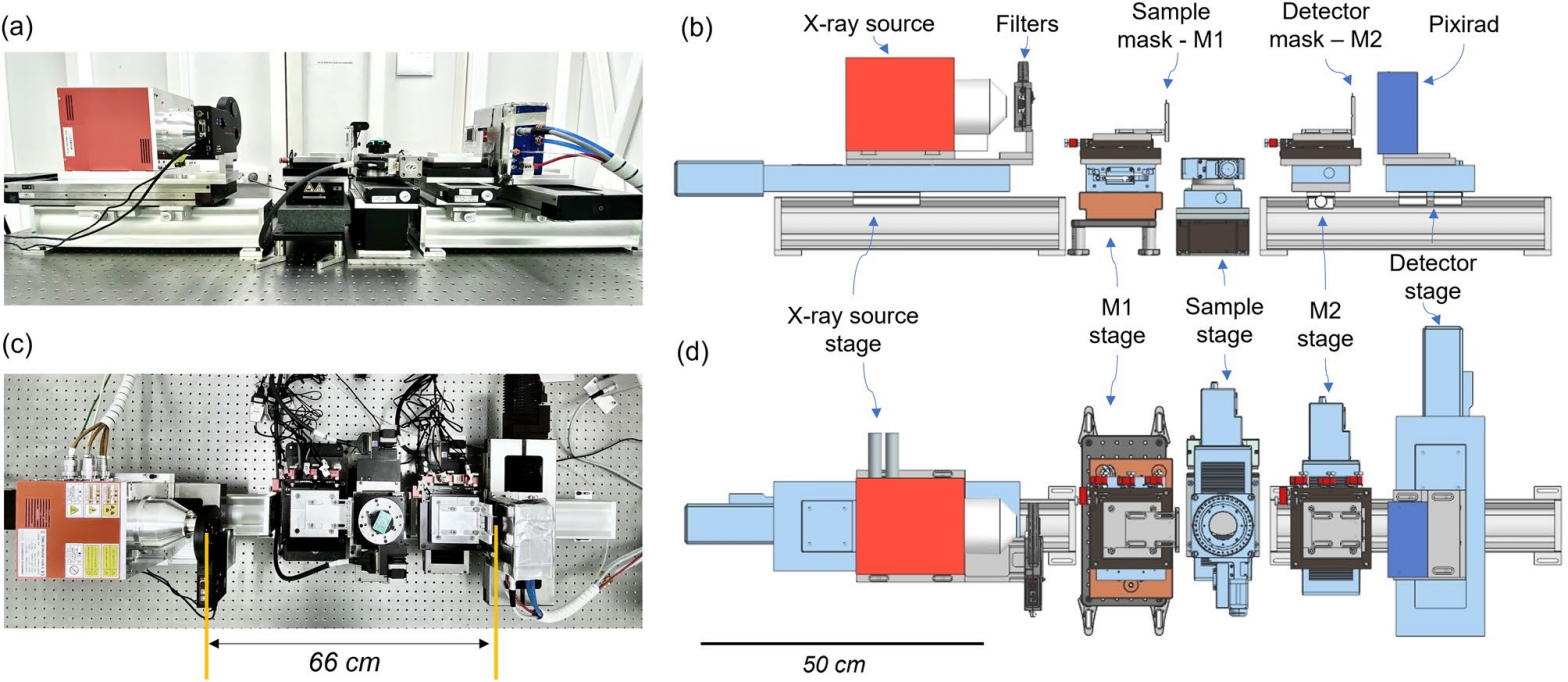
Lateral (**a**) and top (**c**) view photographs of the setup and their respective CAD drawings (**b**,**d**).

Photographs and drawings of the PEPI Lab setup are shown in Fig. 5. To pursue the required flexibility, the implemented setup comprises 5 motion stages, allowing independent motions of X-ray source, sample mask, sample, detector mask, and detector, respectively.

The X-ray source is positioned onto a long travel translator (Newport MTM 250) coupled to an aluminum rail through sliding carriages. The rail allows for coarse manual motion, while the translator is used for fine adjustments of the magnification. A 6 slots filter wheel equipped with a lead collimator and aluminum and copper filters of various thicknesses is positioned in front of the X-ray source, allowing for X-ray collimation and flexible spectral shaping.

The sample mask (M1) is attached to a dedicated aluminum frame coupled to a 6 degrees of freedom multi-axis platform (Newport 8095) that is used for mask alignment. All axes are equipped with remotely controllable piezometric motors with a minimum incremental motion of $\sim$20 nm and travel ranges of a few mm. The platform is positioned onto a long-range and high-precision magnetic drive linear translator (Newport XMS 100), ensuring an accuracy better than 1 $\upmu$m and repeatability better than 0.1 $\upmu$m over a 100 mm travel range. This motor is the key motion element of the setup since it enables precise and fast M1 movements required for the IC stepping, plus it allows the mask to slide out from the X-ray beam when the system is used in XSI mode. To ensure optimal positioning performance, the translator is attached to a dedicated granite base positioned onto an optical breadboard.

The sample stage features a motorized rotator (Newport URM 100), enabling CT, attached to a linear translator (Newport UTM 100), allowing for sample centering and reference (i.e. without sample) flat-field acquisitions. Additionally, the translator is used during EI acquisitions matching the M1 motion during IC stepping, and performing sub-mask pitch motions to provide adequate spatial sampling (i.e. dithering). It should be remarked here that this translator has less demanding performance requirements with respect to the M1 translator as the final image spatial resolution is limited by the mask aperture ($\sim$10 $\upmu$m), therefore not requiring sub-$\upmu$m precision. Moreover, in CT acquisitions, this translator is also used to mitigate ring artifacts in the final image through the jittering procedure, where the sample is slightly displaced at each projection^24^. These motors are positioned onto a vertical translator (Thorlabs MLJ-150) with a 50 mm travel range allowing to scan samples with different heights and to perform multiple vertical steps acquisitions.

The detector mask (M2) is fixed on a frame and positioned onto a multi-axis platform analogous to the one of the sample mask. Differently from M1, once aligned, M2 does not require any motion during acquisition, therefore not posing any strict requirement on motion stages. The platform is coupled to a 100 mm travel range motorized linear translator (Newport UTM 100), allowing to move the mask out when the system is used in single-mask EI or XSI modes. The M2 stage is mounted onto a carriage that can slide over an aluminum rail along the X-ray propagation direction, thus allowing for a coarse matching of M2 and detector pixel pitches.

The detector is mounted onto a 200 mm travel motorized linear translator (Newport MTM 200) allowing both alignment and the implementation of the extended field of view CT modality. The latter consists of a lateral displacement of the detector with respect to the sample’s center of rotation. This, if the acquisition is performed on 360 degrees and a dedicated reconstruction algorithm is applied, allows to laterally increase the FOV by up to 60%^42^.

X-rays are generated by a fixed tungsten anode microfocus tube (Hamamatsu L10101) operating at voltages between 40 and 100 kVp and currents between 10 and 200 $\upmu$A. The experimentally characterized focal spot dimension is in the range from 5 to 30 $\upmu$m and it scales linearly with the requested power (from 5 $\upmu$m at 4 W up to 30 $\upmu$m at 20 W). The imaging detector is a Pixirad-1/Pixie-III photon-counting device^25^, featuring a 650 $\upmu$m thick CdTe sensor bump bonded on a 512$\times$402 matrix of pixels with a size of 62 $\upmu$m, corresponding to an active area of 31.7$\times$24.9 mm^2^. The detector features two independent energy thresholds allowing the acquisition of two images over different energy bins in a single shot, therefore enabling spectral imaging. The onboard electronics are equipped with a charge-sharing compensation mechanism, thus reducing cross-talk between pixels, improving spectral performance, and leading to a pixel-limited point-spread-function^29^. A summary of the system specifications can be found in Table 1.

**Table 1:** PEPI Lab specifications. .

X-ray source	Spectrum (kVp)	40–100
	Power (W)	20 (max.)
	Focal spot ($\upmu$m)	5–30
Masks	Substrate (mm)	1, graphite
	Material ($\upmu$m)	250, Au
	M1 pitch/aperture ($\upmu$m)	61/10
	M2 pitch/aperture ($\upmu$m)	116/19
Detector	FOV (pixels)	$512 \times 402$
	Pixel size ($\upmu$m)	62
	Sensor ($\upmu$m)	650, CdTe
Geometry	Dimension (cm)	66 (EI); 50–120 cm (XSI)
	Magnification	1.8 (EI); 1.3–2.8 (XSI)
	Resolution-PSF ($\upmu$m)	10 (EI, min.); 20 -50 (XSI)

### Software

An in-house control software has been developed for the PEPI Lab, named PEPIControl. The software aims at supporting lab users to let them prepare custom acquisition scripts. Thanks to its popularity within the scientific community and in order to make productive use of open-source freely available software components, PEPIControl has been designed using Python programming language. Users just need to import the PEPIControl library in their Python scripts or notebooks and the relative/absolute positions of all the motors can be set via intuitive commands. Similarly, the X-ray source and the detector are controlled via simple commands. This Python-based software layer has been designed to support hardware variations. Future upgrades of the instrumentation do not require alteration of users’ script: PEPIControl is responsible to adapt its interface (the exported commands) to the new hardware.

A custom graphical user interface (GUI) based on the PyQT framework for the so-called “live” mode of the detector has been also developed. This user interface is tailored for the spectral and phase-contrast applications investigated at PEPI Lab. The effects of basic settings such as e.g. source voltage and current selection, detector exposure time and energy thresholds can be quickly tuned thanks to the “live” GUI.

PEPIControl includes also refined image processing solutions recently developed to compensate for detector imperfections. These solutions include e.g. despeckle image filtering^43,44^, common flat field correction, and artifacts compensation. More interestingly, due to the long scanning time required by some computed tomography protocols, PEPIControl has been designed to support online image processing. This means that while a new tomographic projection is acquired, the previously acquired one is added to a processing queue where all the projection-domain operations are performed. This allows for a speed-up of the post-acquisition steps.

Several support software tools have been also developed for PEPILab. Specifically, the tools serving for mask alignment as well as absorption, refraction, and scattering signal retrieval implement pixel-by-pixel Gaussian fitting procedures, implying hundreds of thousand of fits to be performed as fast as possible. To this aim, a software solution considering a GPU-accelerated Gaussian fitting tool (GPUfit^45^) is currently adopted. Thanks to this support, typically, lab users can perform mask alignment (see next section) and preview processed data in a few minutes. When performing CT experiments, tomographic reconstruction is performed by using the TIGRE^46^ cone-beam reconstruction library. Post-reconstruction processing, typically needed in spectral imaging setting, is also performed via in-house software also including dedicated de-noising and material decomposition algorithms^26^.

### Mask alignment and stability

**Figure 6:**
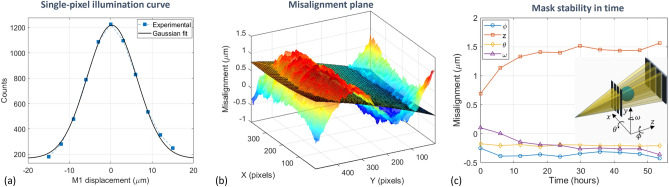
In (**a**) a plot of a single-pixel illumination curve (blue points) and the corresponding Gaussian fit (black line). In (**b**) an example of a misalignment plane (color surface) acquired in double-mask modality, together with the second-order polynomial 2D fit (meshed surface) used to determine positioning misalignment. In (**c**) the positioning misalignment as a function of time where all the components (angles and direction) are shown individually.

Sample mask alignment in EI, either with the detector mask or with the detector pixel matrix, is crucial for extracting phase signals. In practice, alignment is reached when the IC is “in phase” across the whole FOV, i.e. when all pixels are on the same working point on the IC. Following an established procedure^47^, alignment is evaluated pixel-by-pixel by computing the IC central position through Gaussian fitting, as depicted in Fig. 6a. Then misalignment is locally estimated as the variation of the IC central positions across the FOV. Two sources of misalignment can be identified, the first being the mispositioning of the mask along the 3 Cartesian angles ($\phi$, $\theta$, $\omega$) or along the beam direction (*z*), as depicted in the inset of Fig. 6c, and the second being attributed to systematic effects such as imperfections in the mask(s). Mispositioning can be estimated by performing a second-order polynomial 2D fit, where each fit parameter reflects the misalignment along a specific angle/direction that can therefore be corrected by acting on the multi-axis positioning stage, while the residual misalignment is attributed to systematic effects^47^. Figure 6b shows a typical misalignment surface obtained at PEPI Lab in the double-mask configuration. From the polynomial fit, a misalignment in the order of 0.7 $\upmu$m can be estimated across the whole FOV, while the residual systematic misalignment is in the order of 1 $\upmu$m, the latter value being comparable with values reported in the literature for state-of-the-art EI systems^47^. At PEPI Lab, alignment is usually checked and/or performed after each modality switch. Considering that image acquisition and analysis require around 3 minutes and that for cross-checking the alignment procedure is repeated twice, the system is typically aligned in less than 10 minutes.

Along with alignment accuracy, its stability in time is key when performing long measurements such as CT scans. To assess system stability, a 54-hour long scan has been performed where alignment has been checked at 10 equally-spaced time points. The positioning misalignment as a function of time is shown in Fig. 6c, where each misalignment component is shown individually. From the plot, it can be seen that the system’s initial alignment is preserved within 0.4 $\upmu$m when considering the Cartesian angles, while a drift in the order of 0.8 $\upmu$m is seen along the *z* direction. This effect, whose magnitude is comparable to the initial misalignment , can be considered tolerable and can be compensated through image normalization^48^.

## Results and discussion

In this section, some imaging examples acquired with the developed setup, one for each imaging modality depicted in Fig. 2, are presented.

**Figure 7:**
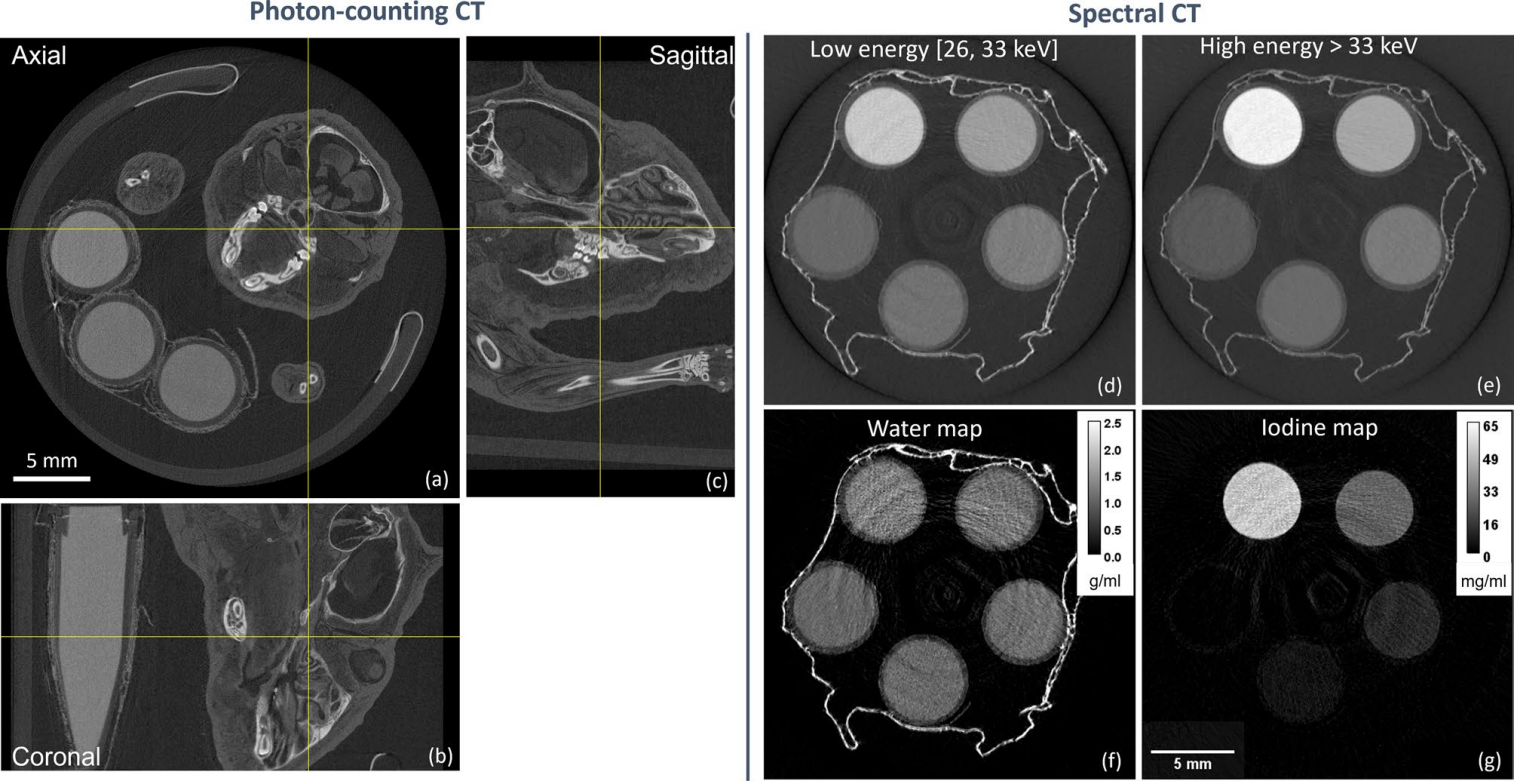
Axial (**a**), coronal (**b**), and sagittal (**c**) views of a plasticized mouse sample acquired in photon-counting CT mode; cuvettes filled with water-based solutions have been used for calibration purposes. In (**d**–**g**) a slice of an iodine-based sample acquired in XSI mode corresponding to the low (**d**) and high (**e**) energy bins and its material-based decomposition into water (**f**) and iodine (**g**) density maps.

### Photon-counting CT

When both masks are out and the detector is operated in single-threshold mode, the setup enables photon-counting attenuation-based imaging. This modality is typically used either for standard CT or for preliminary assessment of the sample (scout) prior to XSI or EI imaging. An example of photon counting CT on a biological sample is shown in Fig. 7 where axial (a), coronal (b), and sagittal (c) views of a plasticized murine sample (PlastiMouse by Smart Scientific Solutions) are displayed. The scan was performed at a voltage of 70 kVp, current 200 $\upmu$A, an added filtration of 0.12 mm of copper and 0.20 mm of aluminum, by acquiring 1440 projections over 360 degrees with an exposure time of 8 s, a photon-counting energy threshold of 26 keV and by using the charge-sharing compensation mode. Magnification was adjusted to 1.37, yielding a 45$\times$45$\times$45 $\upmu$m^3^ voxel, and the acquisition was obtained in the extended field of view mode yielding a final reconstructed volume of 652$\times$652$\times$402 voxels. It should be noted that, compared to conventional micro-CT systems based on indirect conversion detectors, photon-counting devices allow for a complete rejection of electronic noise (i.e. image noise is Poisson dominated) and they detect a non-spectrally-weighted signal, therefore bringing to an increase of contrast-to-noise ratio^13^.

### Spectral CT

By switching the detector to the two-thresholds mode, the implemented setup enables spectral CT imaging. To demonstrate the system’s performance a sample made of 5 plastic cuvettes containing water-iodine solutions with iodine concentrations of 50, 25, 10, 5, 0 mg/ml was imaged. The acquisition was performed with a 50 kVp, 200 $\upmu$A spectrum filtered with 0.12 mm of copper, while the detector thresholds were set to 26 keV and 33 keV, respectively. The tomographic dataset consisted of 1440 equally-spaced projections over 360 degrees with an exposure time of 25 s per projection. By comparing the reconstructed low and high-energy images, shown in panels (d) and (e) of Fig. 7, a signal increase at high energy in the topmost cuvette (highest iodine concentration) can be observed, due to the rise of iodine attenuation coefficient corresponding to its K-edge energy (33.2 keV). By applying a custom decomposition algorithm, including the actual detector energy response^29,30^ (see panel (d) of Fig. 2) and a deep learning-based denoising algorithm^26^, quantitative water and iodine density maps are obtained, as displayed in panels (f) and (g) of Fig. 7. By measuring mean and standard deviation values of a circular ROI within each cuvette, experimental densities and respective uncertainties were determined to be 49 ± 4, 25 ± 3, 10 ± 2, 5 ± 2, and 0 ± 2 mg/ml. These values are compatible with nominal densities and demonstrate the system’s quantitativeness and sensitivity at the level of a few mg/ml with a reconstructed voxel size of 35$\times$35$\times$35 $\upmu$m^3^. The correctness of the decomposition is also supported by the water image, where no relevant iodine signal contamination is observed.

### Phase-contrast double-mask EI

**Figure 8:**
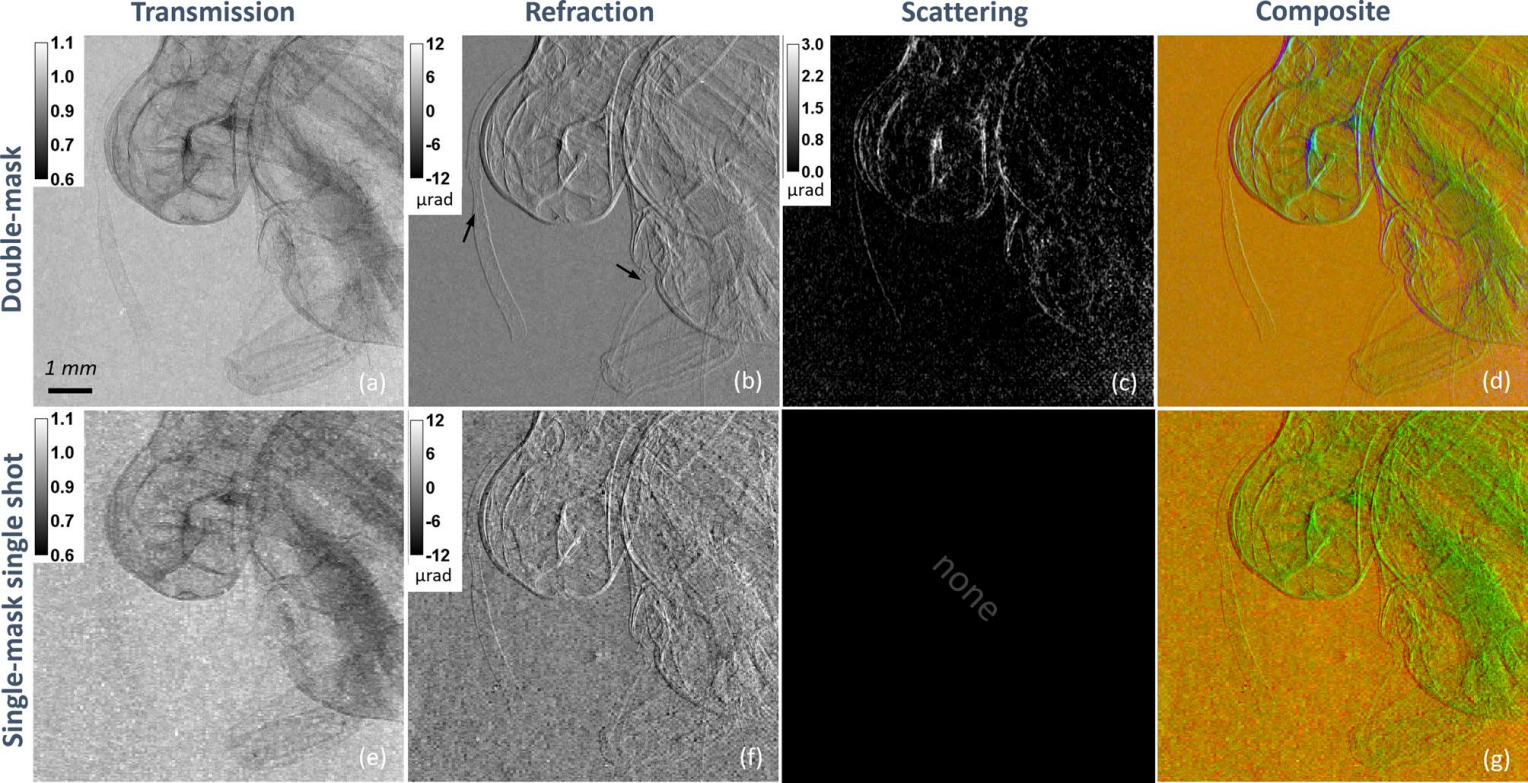
Transmission (**a**), refraction (**b**), and scattering (**c**) image of a violet carpenter bee (*Xylocopa violacea*) acquired in double-mask mode. The composite image in (**d**) is an RGB image with transmission (red), refraction (green), and scattering (blue). In (**e**,**f**) the same sample acquired in single-mask single-shot mode. In (**g**) the respective composite image with transmission (red) and refraction (green) channels. Arrows in (**b**) point to subtle structures within the antenna and wing that are visible only in the refraction image.

The double-mask EI configuration requires sliding both masks within the FOV. To test the system’s phase-sensitivity on a biological sample, an *ex-vivo* violet carpenter bee (*Xylocopa violacea*) specimen has been imaged. Data have been collected by using a 60 kVp spectrum, no added filtration, and a current of 150 $\upmu$A. The scan consisted of 11 sampling points on the IC, 8 dithering steps, and 6 s per exposure. By applying a pixel-wise Gaussian fitting^45^ along the IC curve steps, the three independent signals, namely attenuation/transmission, refraction, and scattering, have been extracted as shown in Fig. 8a–c. The refraction image, in panel (b), clearly shows the sample’s sharp interfaces, revealing details, e.g. within the animal’s antenna and wing (see arrows), that are scarcely visible in the attenuation channel, in panel (a). The scattering image, in (c), mainly highlights regions in the bee’s head corresponding to some more complex structures, arguably containing multiple interfaces. The fusion of the images into a composite color image Fig. 8d gives the idea of the complementarity of information contained in the three channels. Refraction images can be used to estimate the angular sensitivity of the system^27^. By measuring the noise in 5 non-overlapping ROIs in the background region of panel (b), sensitivity is estimated to be 0.59±0.02 $\upmu$rad. This value is in line with the angular sensitivities of other XPCI systems making use of microfocus X-ray sources and similar acquisition times. For instance in grating interferometry Thüring et al.^49^ reported values of 0.55 $\upmu$rad (for a 60 kVp spectrum); in speckle-based imaging Quenot et al.^50^ reported 5 $\upmu$rad while Zanette et al.^7^ 0.24 $\upmu$rad (by using a liquid metal jet source); in EI Navarrete-Leon et al.^51^ reported 0.35 $\upmu$rad. In this context, it should also be noted that a significant deviation from the mentioned sensitivities in the fraction of $\upmu$rad range, has been recently reported by Vila-Comala et al.^52^ where, through the use of a microstructured anode microfocus source, sensitivities down to 0.05 $\upmu$rad were achieved with a grating interferometry setup.

**Figure 9:**
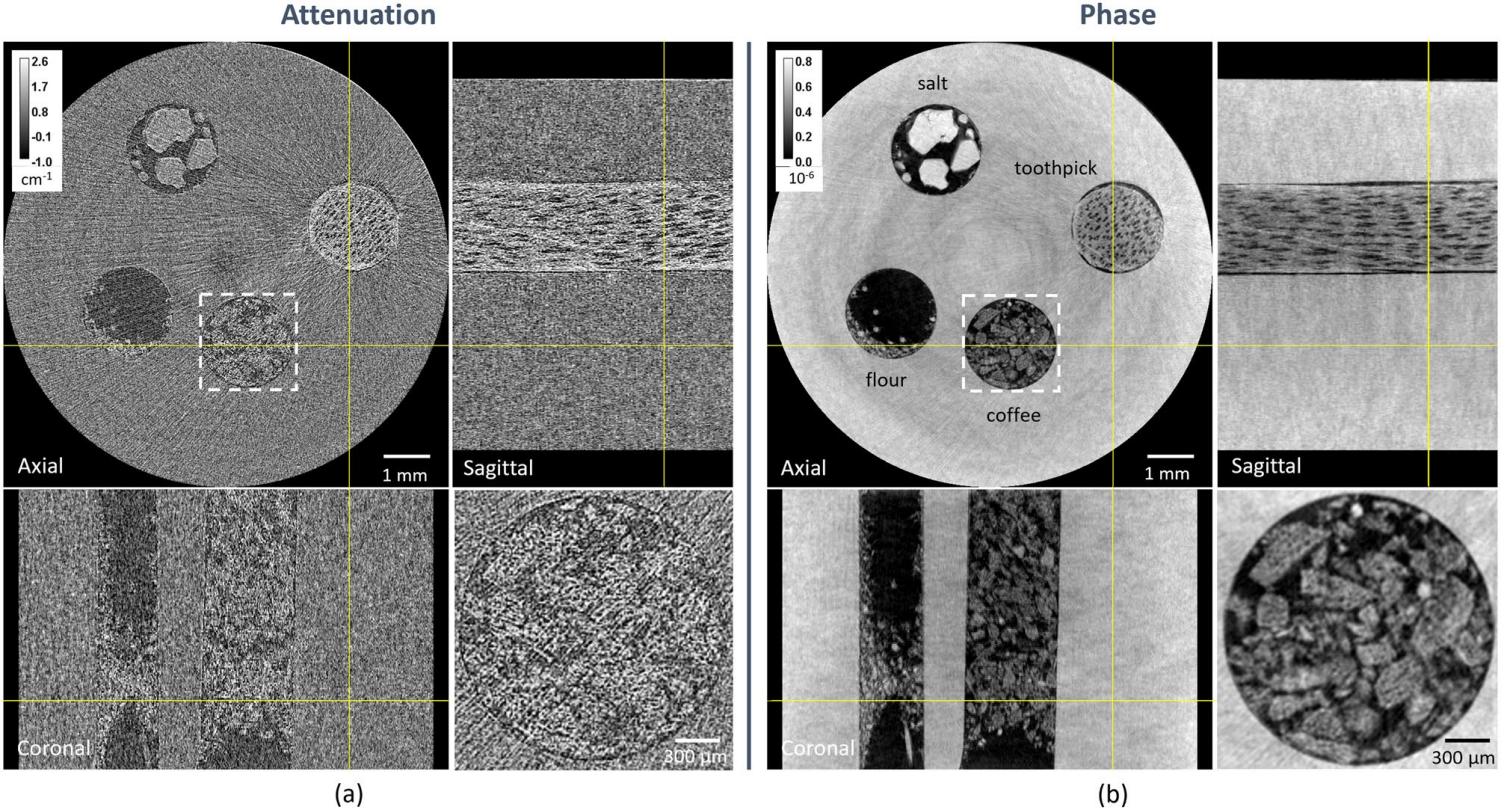
CT images of the plastic phantom with inserts acquired in the double-mask imaging modality. In (**a**,**b**) axial, sagittal, and coronal views of the attenuation and (integrated) phase channels, respectively. Dashed boxes in the axial views identify the zoomed-in coffee powder detail of shown at the bottom-right of each panel.

CT capabilities have also been tested by imaging a test object consisting in a cylindrical PMMA holder with 4 holes, whereby 4 commonly used light materials, i.e. kitchen salt, flour, coffee powder, and a toothpick, were inserted. The dataset consists of 720 projections acquired at 50 kVp and 150 $\upmu$A, with an exposure time of 4.5 s, 5 positions on the IC, and 6 dithering steps corresponding to an acquisition time of 27 h. Reconstructed attenuation and (integrated) phase axial, sagittal, and coronal views of the sample are shown in Fig. 9. The phase channel yields a higher image quality, bringing to a 15-times higher SNR (measured on the PMMA holder), and generally a better detail, as can be seen for instance from the fine structure of coffee powder in the axial view (see zoomed-in detail).

### Phase-contrast single-mask EI

By sliding out the detector mask and aligning the sample mask such as the unperturbed beamlets impinge in between every other pixel column, the system is in the single-mask EI configuration. As detailed in the previous section, this modality enables single-shot phase imaging and no mask motion is required during the acquisition. On the other hand, if a high spatial resolution is required, dithering is still needed to provide an adequate sampling of the imaged object. With the aim of performing a reasonable comparison, the same detail of the bee sample imaged in the double-mask configuration has been acquired. Acquisition parameters were kept constant (60 kVp, 150 $\upmu$A, 6 s exposure time, and 8 dithering steps) but only one position of the mask (single shot), instead of 11, was used, therefore reducing the overall exposure by 91%. The resulting transmission and refraction channels are shown in Fig. 8e–f, while the composite is reported in (g). By comparing the images with the double-mask case it can be seen that the physical image content is the same while a higher noise is observed in single-mask mode. This is reflected in refraction sensitivity, which is estimated to be 1.77±0.15 $\upmu$rad. It should be noted that the increased noise is consistent with the 11-fold exposure reduction under the assumption of Poisson statistics. Additionally, it should be mentioned that no scattering channel can be retrieved from a single-shot single mask acquisition. Overall, this configures the use of single-mask modality for applications less demanding in terms of phase-related signal extraction and requiring faster scans, hence higher throughput.

## Conclusions and future outlook

In this work, we introduced a new flexible multi-modal tabletop system enabling photon-counting attenuation-based, spectral, and phase-contrast imaging, both in planar and tomographic geometries. The most relevant steps in the design, simulation, and implementation have been outlined with the aim of showing a path from the initial idea to the realization of a compact imaging system. Specifically, based on a dedicated Geant4 simulation platform, the effect of many system parameters namely X-ray source size, overall dimension, thickness, substrate, and aspect ratio of masks has been studied, therefore guiding the system implementation. Albeit preliminary, the results presented in this work demonstrate that the system fulfills its design requirements while being stable in time (misalignment below 1 $\upmu$m across the FOV during 54 h of monitoring). Specifically, in addition to photon-counting CT of a biologically relevant sample, spectrally decomposed CT images of an iodine-based sample have been presented, demonstrating a contrast medium sensitivity below 5 mg/ml. Both single-mask and double-mask EI phase-contrast modalities have been implemented and tested on a biological sample. The double-mask configuration demonstrated a refraction sensitivity of 0.59 $\upmu$rad, which is in line with other XPCI compact systems based on off-the-shelf microfocus sources, while the single-mask single-shot configuration reached sensitivity in the order of 1.8 $\upmu$rad by using less than 1/10 of the exposure. Phase CT capabilities have also been assessed on a plastic phantom containing light materials, showing a 15-fold SNR improvement compared to the attenuation channel.

This work represents the first step of the new system. In fact, also as a function of the specific application, an experimental optimization of acquisition parameters such as X-ray spectra, illumination curve stepping, and dithering will be carried out to extract the best performance. Finally, it should be remarked that reducing scan times for phase-contrast CT from several (>10) to a few hours is one of the main challenges within the community developing XPCI micro-CT systems. In this context, in addition to the mentioned optimization, we will implement the cycloidal tomography acquisition scheme^53^, whereby dithering steps can be reduced while preserving spatial resolution in CT scans, and work on the integration of phase-contrast specific deep-learning de-noising filters, that we have already successfully applied to conventional and spectral data^26^.

## Data Availability

Data presented in this work are available upon reasonable request by contacting the corresponding author.
